# Genetically diverse *Plasmodium falciparum* infections, within-host competition and symptomatic malaria in humans

**DOI:** 10.1038/s41598-018-36493-y

**Published:** 2019-01-15

**Authors:** Paul Sondo, Karim Derra, Thierry Lefevre, Seydou Diallo-Nakanabo, Zekiba Tarnagda, Odile Zampa, Adama Kazienga, Innocent Valea, Hermann Sorgho, Jean-Bosco Ouedraogo, Tinga Robert Guiguemde, Halidou Tinto

**Affiliations:** 1 Institut de Recherche en Sciences de la Santé (IRSS)/Clinical Research Unit of Nanoro (CRUN), Nanoro, Burkina Faso; 2 MIVEGEC, IRD, CNRS, University of Montpellier, Montpellier, France; 3 Centre Muraz of Bobo-Dioulasso, Bobo-Dioulasso, Burkina Faso

## Abstract

There is a large genetic diversity of *Plasmodium falciparum* strains that infect people causing diverse malaria symptoms. This study was carried out to explore the effect of mixed-strain infections and the extent to which some specific *P. falciparum* variants are associated with particular malaria symptoms. *P. falciparum* isolates collected during pharmacovigilance study in Nanoro, Burkina Faso were used to determine allelic variation in two polymorphic antigens of the merozoite surface (*msp1* and *msp*2). Overall, parasite density did not increase with additional strains, suggesting the existence of within-host competition. Parasite density was influenced by *msp1* allelic families with highest parasitaemia observed in MAD20 allelic family. However, when in mixed infections with allelic family K1, MAD20 could not grow to the same levels as it would alone, suggesting competitive suppression in these mixed infections. Host age was associated with parasite density. Overall, older patients exhibited lower parasite densities than younger patients, but this effect varied with the genetic composition of the isolates for the *msp1* gene. There was no effect of *msp1* and *msp*2 allelic family variation on body temperature. Haemoglobin level was influenced by *msp*2 family with patients harboring the FC27 allele showing lower haemoglobin level than mono-infected individuals by the 3D7 allele. This study provides evidence that *P. falciparum* genetic diversity influenced the severity of particular malaria symptoms and supports the existence of within-host competition in genetically diverse *P. falciparum*.

## Introduction

Malaria is the commonest parasitic disease worldwide leading to about 445000 deaths annually^1^. Among *Plasmodium* species infecting humans, *Plasmodium falciparum* is the most prevalent and the most dangerous, responsible for over 90% of deaths due to malaria^2^. This species is also the most affected by resistance to antimalarial drugs, which constitutes a major challenge in the fight against malaria. Moreover, there is great genetic polymorphism within *P. falciparum* species and people living in endemic area are frequently and simultaneously infected by several *Plasmodium* strains^3^. This genetic diversity of the parasite is one of the main factors responsible for the slow acquisition (several years) of immunity against malaria. Thus, individuals must meet the entire diversity of circulating parasite populations before they develop an effective anti- malarial immunity^3^. This genetic polymorphism of the parasite constitutes also a challenge for the development of an effective anti- malarial vaccine^4^. At the same time, there is a large variability of the clinical manifestations of *P. falciparum* malaria^5^. There are no specific symptoms for *P. falciparum* malaria infection which limits the accuracy of clinical diagnosis. Clinical diagnosis constitutes to date a common practice in poor resource settings due to frequent stock out of rapid diagnostic tests (RDTs)^5^. However, it is well established that the risk for an individual to fall clinically ill with malaria results from a complex interaction between the parasite, environment and human host^6,7^. Considering the factors related to the parasite, the existence of variation in strain virulence has been suggested by several authors^8,9^. In animal models, strains of malaria parasite have shown significant differences in virulence and pathogenicity^10,11^. However the use of these experimental infection methods to directly estimate the level of strain virulence has always been limited in human malaria for obvious ethical reasons. Nevertheless, in *P. falciparum* some level of virulence can be assessed indirectly by correlating clinical manifestations of the disease with the biological characteristics of the circulating parasites^12–15^. Accordingly, associations between some *P. falciparum msp*-variants and the occurrence of clinical malaria, severe anemia and cerebral malaria were previously reported^9,12,13,16–20^.

Besides associations between specific allelic variant of the parasite and particular disease symptoms, the presence of multiple competing clones within a host can also affect disease severity^21,22^. When different clones share the same environment (the host and its immune response) and use the same resource (the host red blood cells), the intrinsic growth rate of each clone can be limited by the presence of other clones; that is, within-host competition occurs^21^. Most evidence for the existence of such within-host competition comes from controlled coinfections experiments in the mouse malaria model, *P. chabaudi*^23–31^. In human malaria, several correlational epidemiological studies support the existence of within-host competition, such that densities of individual parasite genotypes are suppressed when other genotype are present^31–33^. Therefore, within-host competition could affect host health through effects on total parasite densities or through the modification of the densities of more or less virulent clones^21^. Accordingly, previous studies found that within-host genetic diversity was associated with disease severity (positively^34,35^; or negatively^31,36^). In addition, the rising clone multiplicities of *Plasmodium falciparum* infections increased the likelihood of clinical malaria episodes as well as the severity of the disease^12,37–39^.

Although many polymorphic antigens have been described in several stages of the parasite life cycle, merozoite surface protein 1 and 2 (*msp1* and *msp*2) seem to be the most appropriate to distinguish parasite populations^40–42^. This study was carried out using these polymorphic markers to explore: (i) whether some specific family strains of *P. falciparum* were mostly associated with the occurrence of particular malaria symptoms such as high body temperature, severe anemia, and high parasite densities; (ii) whether within-host competition occurred (when certain genotypes in mixed infections cannot grow to the same levels as it would alone); and (iii) whether mixed infections were associated to lower or higher disease severity.

## Methods

### Study area and population

The study was carried out in Nanoro health district (NHD) located in the central part of Burkina Faso (West Africa). Data collection was performed at two peripheral health facilities (Nanoro and Nazoanga). The area is characterized by an alternation of two distinct seasons: a rainy season occurring from June/July to October/November in which malaria transmission is highest, followed by a long dry season from November to May. *P. falciparum* is the commonest malaria parasite and its transmission is mainly assured by *Anopheles gambiae s.l*. and *Anopheles arabiensis* complex^43^. In 2014, the population size was estimated at 158127 inhabitants and is composed of mossi, gourounssi and fulani ethnic groups respectively^44^.

### Samples and study design

*P. falciparum* isolates were obtained from patients enrolled in a larger pharmacovigilance trial aimed at assessing the effectiveness of Artesunate-Amodiaquine *versus* Artemether-Lumefantrine in Nanoro, Burkina Faso. Details of methodology and results of this clinical trial are published elsewhere^45^. The present study reports on screened patients including: (i) all those who were included for assessment of the effectiveness of the two drugs (Uncomplicated malaria group, n = 675) and (ii) those classified as severe malaria (n = 52) and were excluded during the effectiveness trial i.e. those with hyper-parasitaemia [Parasite density (DP) ≥ 200000 asexual forms/µL] and severe anaemia [Haemoglobin level (Hb) ≤ 5 g/dl]. Patients were physically examined before enrolment. History of the disease and different symptoms were reported on the case record form (CRF). Blood samples were taken for microscopic examination, haemoglobin (Hb) measurement, and spotted onto Whatman filter papers which were air dried and stored in plastic bags with silica gel for later PCR analysis. Two markers of *P. falciparum* genetic polymorphism were used to differentiate recrudescence and new infection: merozoite surface proteins 1 and 2 (*msp1* and *msp*2). The present report used data and samples collected on Day 0 (before enrolment) to seek correlation between the presence of *msp1* and *msp2* allelic families and parasite virulence. Three surrogates of parasite virulence and pathogenicity were used: (i) hyperthermia (high body temperature), (ii) anemia (low haemoglobin level) - two host traits corresponding to objectively measurable malaria signs - and (iii) parasitaemia (parasite density), an important parasite trait.

### Determination of axillary temperature, parasite density and Hb level

Axillary temperature was determined before enrolment using an electronic clinical thermometer. The tip of the thermometer was inserted under the armpit for both children and adult and numeric value of axillary temperature was recorded.

Screening for anemia was performed by the measurement of Hb level. A HemoCue® 301^+^ was used for photometric measurement of Hb in the field. Undiluted blood was drawn into the cavity of the microcuvette which was inserted into the analyzer and Hb concentration was obtained in g/dL immediately.

Parasite density was determined by counting the number of asexual parasites per 200 white blood cells, and calculated per micro liter of blood by assuming the white blood cells at 8,000 per µL. A smear was declared negative when the examination of 100 thick-film fields did not reveal the presence of asexual parasites.

### Parasite genotyping

Molecular analysis was performed at the laboratory of Centre Muraz in Bobo-Dioulasso, Burkina Faso. *P. falciparum* DNA was extracted from dried blood spots using the Qiagen DNA extraction Kit (QIamp, Qiagen, Germany) method and 80 µL of DNA template was obtained.

*msp1* and *msp2* allelic families were genotyped in a nested PCR approach as previously described^46^. Briefly, an initial amplification of the outer regions of the block 2 of *msp1* and block 3 of *msp2* was performed. The second round used the first PCR product as DNA template with the specific primers of each of the allelic family of the two genes. After DNA amplification, 5 µL of PCR product were used for visualization of DNA bands in ethidium bromide-stained 2.5% agarose gels for 2 hours at 100 V. Culture strains spotted on filter paper were used as positive controls: 3D7 for *msp1*-K1 and *msp2*–3D7, D10 for *msp1*-MAD20 and *msp2*-FC27 and RO33 for *msp1*-RO33 allelic family. No DNA template obtained from extraction of a sterile filter paper served as negative control.

### Statistical analysis

A sample was considered as belonging to a given allelic family if there was an occurrence of at least one band after DNA amplification using the family specific primers. The prevalence of each allelic family was estimated by calculating the percentage of fragments assigned to this family among the overall number of fragments detected for that locus. Multiplicity of infection (*MOI*) was defined as a mean number of parasite genotypes by clinical isolate. Within-host competition was assessed by considering density as a valuable measure of parasite interaction within an infected human host.

All statistical analyses were performed in R (version 3.0.1)^47^. A Generalized Linear Model (GLM, negative binomial errors) was used to investigate the effect of *msp1* (7 levels: K1, MAD20, RO33, K1 + MAD20, K1 + RO33, MAD20 + RO33, K1 + MAD20 + RO33) and *msp2* (3 levels: 3D7, FC27, 3D7 + FC27) allelic families, host age, and first order interactions on parasite density. For *msp1* allelic family, an additional GLM with a negative binomial distribution was used to explore the effect of multiple infections (4 levels: K1, MAD20, RO33, or mixed) on parasite density. Separate GLMs with Gaussian distributions were used to investigate the effects of *msp1* (7 levels) and *msp2* (3 levels) allelic families, host age, parasite density and first order interactions on axillary temperature and Hb level, respectively (models were checked for homogeneity of variance by using Fligner-Killeen tests)^48^. For *msp1* allelic family, additional gaussian GLMs were used to explore the effect of multiple infections (2 levels: mono *versus* mixed infection) on axillary temperature and Hb level, respectively. Model simplification used stepwise removal of terms, followed by likelihood ratio tests (LRT). Term removals that significantly reduced explanatory power (*p* < 0.05) were retained in the minimal adequate model^48^.

### Ethical Consideration

This is part of a large study entitled pharmacovigilance for artemisinine based combination treatments in Africa. The ethical approval for the study was provided by the institutional Ethics committee of Centre Muraz, Bobo-Dioulasso, Burkina Faso; the National Ethic committee of Burkina Faso and the WHO-TDR Ethics Review Committee. The study was conducted in accordance with laws and regulations in Burkina Faso and international principles of Good Clinical Practices (GCP). Before enrolment a written informed consent was obtained from patients (adults) or parents/guardians (children). For illiterates, in addition to the thumb impression of the participant/parent/guardian, a signature of an impartial witness was obtained.

## Results

During the study period (September 2010–October 2012), 1480 patients were screened, 1010 patients were diagnosed positive for malaria and 980 patients were mono-infected by *P. falciparum*. Out of them, 748 patients had parasitaemia ≥2000 asexual forms per microliter of blood. All available Day 0 blood spots were systematically considered for *msp1* and *msp2* genotyping (n = 727). Unsuccessful PCR concerned one sample in both *msp1* and *msp2* genes. Sex ratio male/female was 1.10 with a median age of 4 (Q25 = 2 - Q75 = 6) years.

Overall, *msp1* and *msp2* genes yielded 1720 and 1660 different fragments respectively. The multiplicity of infection (*MOI*) was 2.3 for *msp1* and 2.2 for *msp2*. Table 1 shows the prevalence of *msp1* and *msp2* allelic families in each of the two study sites.

**Table 1:** Prevalence of *msp1* and *msp2* allelic families by study site.

Gene	Allelic family	Nanoro	Nazoanga	Total	p-value
*msp1*	K1	51.3 (437/852)	51.1 (444/868)	51.2 (881/1720)	0.933
	MAD20	25.9 (221/852)	27.1 (235/868)	26.5 (456/1720)	0.572
	RO33	22.8 (194/852)	21.8 (189/868)	22.3 (383/1720)	0.901
*msp2*	3D7	57.7 (491/851)	58.0 (469/809)	57.8 (960/1660)	0.901
	FC27	42.3 (360/851)	42.0 (340/809)	42.2 (700/1660)	

No difference was found in the prevalence of the different allelic families between the two study sites (X^2^ tests, all five P-values > 0.05). The commonest *msp1* allelic family was the K1 type with a prevalence of 51.2% (881/1720). The most prevalent *msp2* allelic family was the 3D7 type with a prevalence of 57.8% (960/1660). No *msp1* or *msp2* allelic family was overrepresented in uncomplicated or severe malaria groups (Table 2).

**Table 2:** Prevalence of *msp1* and *msp2* allelic families in mild and severe malaria groups.

Polymorphic antigen	Allelic Family	Uncomplicated malaria	Severe malaria	P-value
*msp1*	K1	51.41 (836/1626)	48.94 (46/94)	0.744
	MAD20	26.57 (432/1626)	25.53 (24/94)	0.910
	RO33	22.02 (358/1626)	25.53 (24/94)	0.688
*msp2*	3D7	57.41 (891/1552)	63.89 (69/108)	0.293
	FC27	42.59 (661/1552)	36.11 (39/108)	0.234

### Parasite density

**Figure 1:**
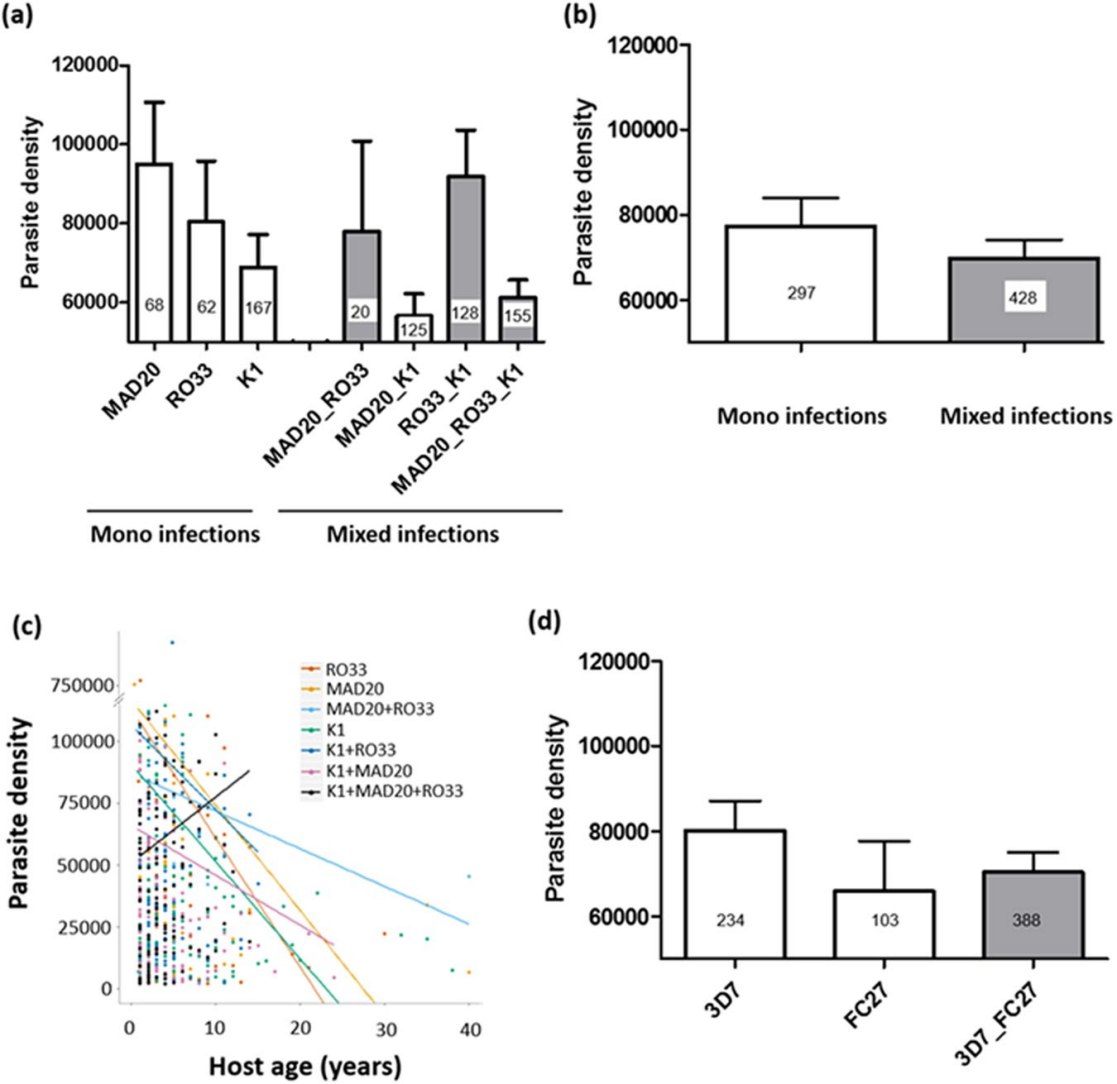
Parasite density (number of asexual parasite stages/µl). (**a**) Effect of *msp1* allelic family variation on parasite density. White bars represent clinical isolates containing single clones of *P. falciparum* at the *msp1* gene (i.e. mono-infections) while grey bars represent clinical isolates containing multiple clones of *P. falciparum* at the *msp1* gene (i.e. mixed-infections). (**b**) Effect of mixed *versus* mono-infection at the *msp1* gene on parasite density. “mono-infections” corresponds to the mean parasite density across RO33, MAD20 and K1 alone (i.e. the three white bars in (**a**)) while “mixed-infection” corresponds to the mean parasite density across K1_RO33, MAD20_RO33, K1_MAD20 and K1_MAD20_RO33 (the four grey bars in (**a**)). (**c**) Effect of host age (in years) on parasite density. Each color line represents a linear relationship fitted to the values of parasite density for each *msp1* allele combination. (**d**) Effect of *msp2* allelic family variation on parasite density. White bars represent clinical isolates containing single clones of *P. falciparum* at the *msp2* gene (i.e. mono-infections) while grey bars represent clinical isolates containing multiple clones of *P. falciparum* at the *msp2* gene (i.e. mixed-infections). Numbers in bars indicate the sample size for each group.

The asexual *P. falciparum* parasitaemia from the collected samples ranged from 2000 to 938750 parasites/μl of blood with a geometric mean (±gsd) of 35814.81 (±3.58). Parasite density was significantly influenced by the *msp1* allelic family with highest parasitemia observed for MAD20, followed by RO33 and K1 (LRT *X*^*2*^_6_ = 22.73, P = 0.0009, Fig. 1a). Clinical isolates containing multiple clones of *P. falciparum* at the *msp1* gene (i.e. multiple infections) displayed similar parasite density as isolates with monoclonal infections (LRT *X*^*2*^_1_ = 1.53, P = 0.22, Fig. 1b), suggesting the existence of within-host competition (Fig. 1b). For MAD20, parasite densities even significantly decreased in mixed infections with K1 (multiple pairwise post-hoc comparisons: MAD20 *versus* MAD20_K1, z = 3.4, P = 0.004; MAD20 *versus* MAD20_RO33_K1, z = 3, P = 0.013, Fig. 1a). This suggests that in those mixed infections, MAD20 cannot grow to the same levels as it would alone, suggesting competitive suppression.

Host age was strongly associated with parasite density with older patients exhibiting lower parasite densities (LRT *X*^*2*^_1_ = 36.4, P < 0.0001, Fig. 1c). This effect varied with the genetic composition of the isolates for the *msp1* gene (*msp1* × Age interaction: LRT X^2^_6_=16.37, P= 0.012, Fig. 1c) . In particular, while parasite density displayed a marginally non-significant increase with age in patients harboring the triple infection K1 + MAD20 + RO33 (P = 0.072), density decreased with age in patients infected with all other combinations. All other interactions were non-significant. No significant differences in parasite density were observed among *msp2* alleles 3D7, FC27 in single or in mixed infections (LRT *X*^*2*^_2_ = 2.12, P = 0.35, Fig. 1d), further suggesting the existence of within-host competition.

### Body Temperature

**Figure 2:**
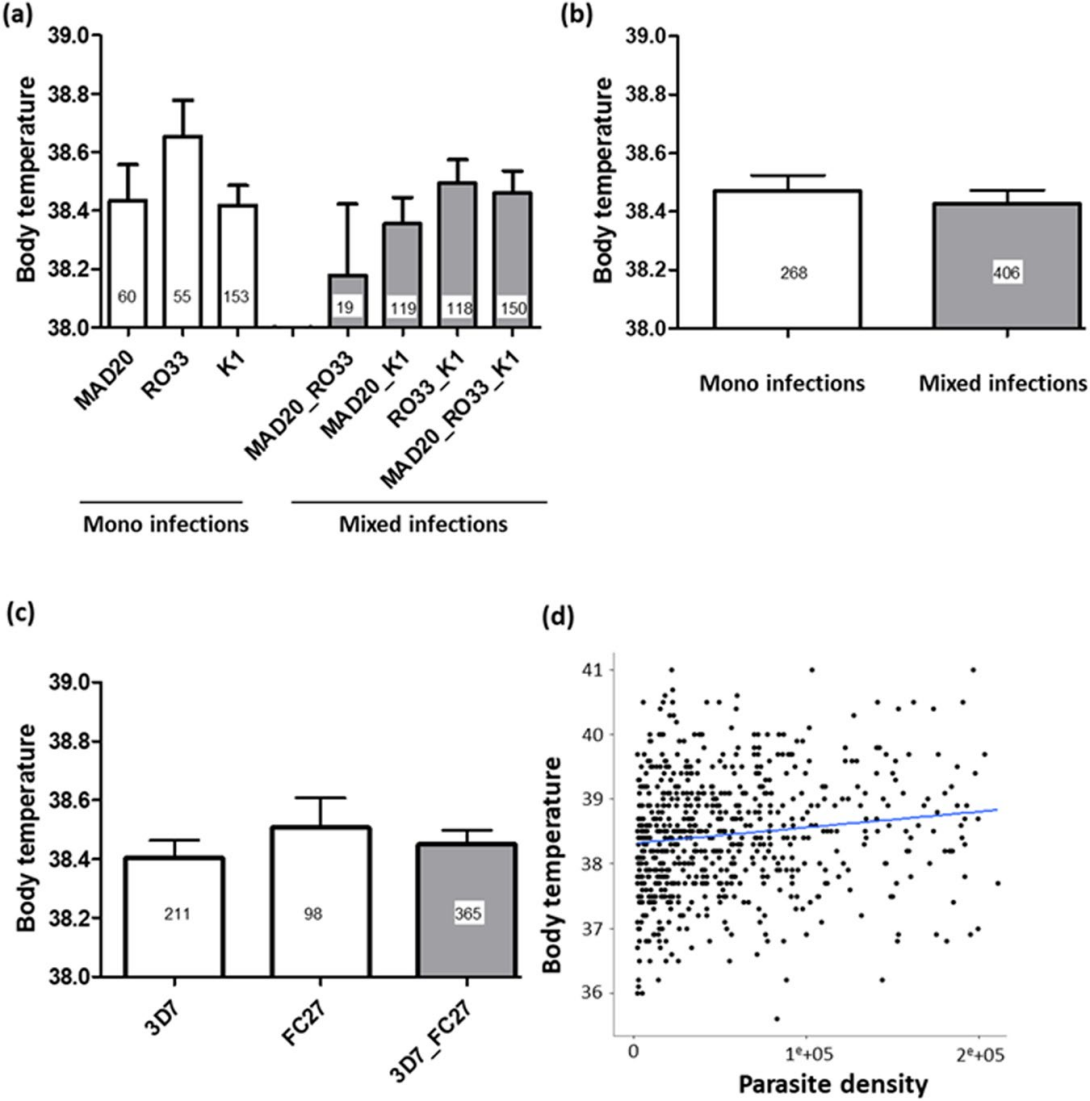
Body temperature (°C). (**a**) Effect of *msp1* allelic family variation on body temperature. White bars represent clinical isolates containing single clones of *P. falciparum* at the *msp1* gene (i.e. mono-infections) while grey bars represent clinical isolates containing multiple clones of *P. falciparum* at the *msp1* gene (i.e. mixed-infections). (**b**) Effect of mixed *versus* mono-infection at the *msp1* gene on parasite density. “mono-infection” corresponds to the mean parasite density across RO33, MAD20 and K1 alone (i.e. the three white bars in (**a**)) while “mixed-infection” corresponds to the mean parasite density across K1_RO33, MAD20_RO33, K1_MAD20 and K1_MAD20_RO33 (the four grey bars in (**a**)). (**c**) Effect of *msp2* allelic family variation on body temperature. White bars represent clinical isolates containing single clones of *P. falciparum* at the *msp2* gene (i.e. mono-infections), while grey bars represent clinical isolates containing multiple clones of *P. falciparum* at the *msp2* gene (i.e. mixed-infections). (**d**) Effect of parasite density on body temperature. The line represents a linear relationship fitted to the values of body temperature. Numbers in bars indicate the sample size for each group.

The mean (±se) axillary temperature was 38.4 (±0.03) °C. There was no significant effect of *msp1* and *msp2* allelic families on axillary temperature (F_6,671_ = 1.13, P = 0.34, and F_2,665_ = 0.70, P = 0.50 respectively, Fig. 2a,c). Parasite isolates containing multiple clones of *P. falciparum* at the *msp1* gene (i.e. multiple infections) did not induce an increase of body temperature compared to isolates with monoclonal infections (F_1,672_ = 0.363, P = 0.55, Fig. 2b). Axillary temperature was significantly influenced by parasite density; the higher the parasitaemia, the higher the temperature (F_1,673_ = 9.86, P = 0.0017, Fig. 2d). Host age was not associated with axillary temperature (F_1,672_ = 1.9, P = 0.17), and there were no significant interactions.

### Hb level

**Figure 3:**
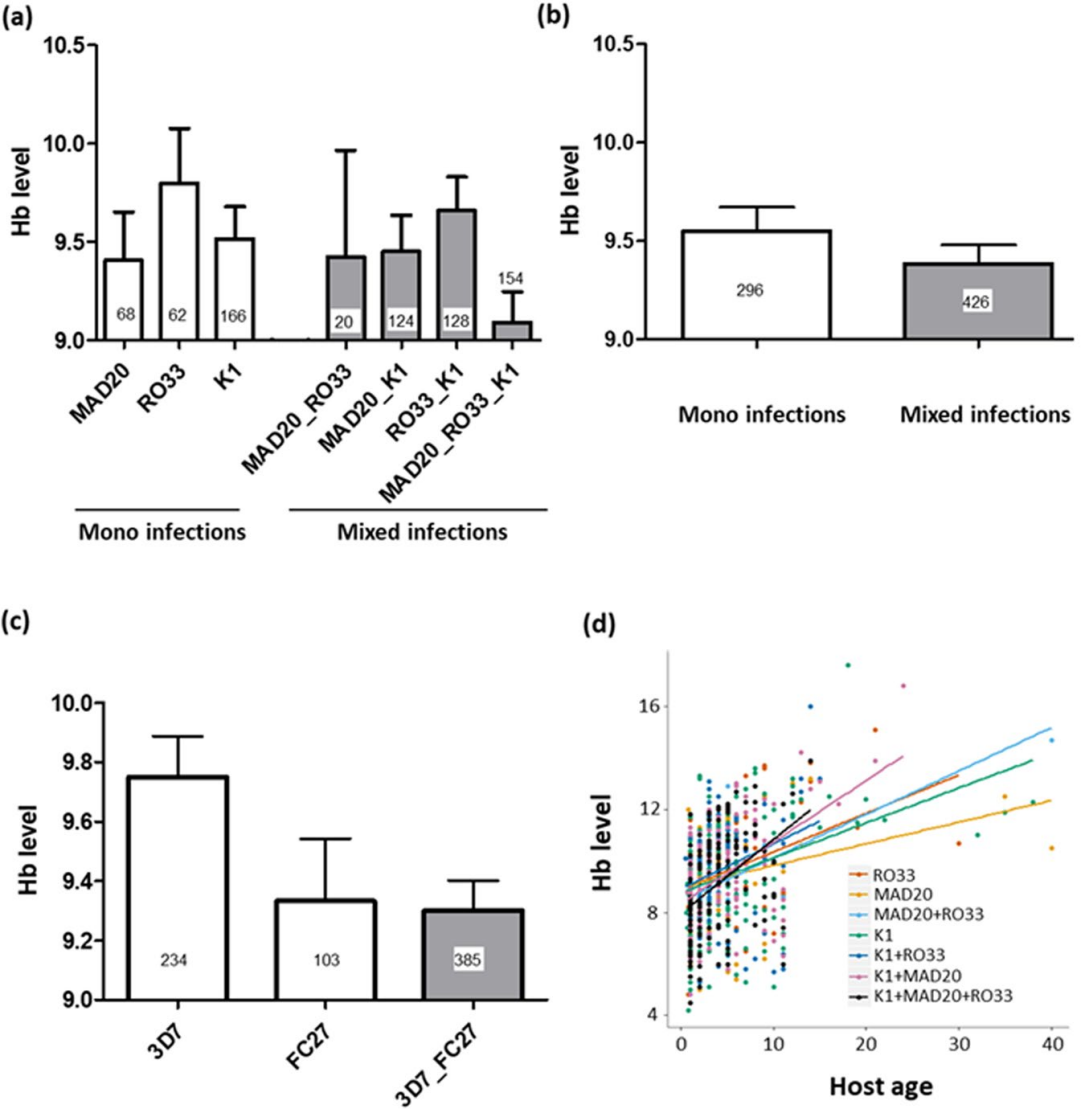
Haemoglobin (Hb) level (g/dL) (**a**) Effect of *msp1* allelic family variation on Hb level. White bars represent clinical isolates containing single clones of *P. falciparum* at the *msp1* gene (i.e. mono-infections) while grey bars represent clinical isolates containing multiple clones of *P. falciparum* at the *msp1* gene (i.e. mixed-infections). (**b**) Effect of mixed *versus* mono-infection at the *msp*1 gene on Hb level. “mono-infection” corresponds to the mean parasite density across RO33, MAD20 and K1 alone (i.e. the three white bars in (**a**)) while “mixed-infection” corresponds to the mean parasite density across K1_RO33, MAD20_RO33, K1_MAD20 and K1_MAD20_RO33 (the four grey bars in (**a**)). (**c**) Effect of *msp2* allelic family variation on Hb level. White bars represent clinical isolates containing single clones of *P. falciparum* at the *msp2* gene (i.e. mono-infections) while grey bars represent clinical isolates containing multiple clones of *P. falciparum* at the *msp2* gene (i.e. mixed-infections). (**d**) Effect of host age on Hb level. Each color line represents a linear relationship fitted to the values of Hb level for each *msp1* allele combination. Numbers in bars indicate the sample size for each group.

The median (Q25–75) Hb level was 9.6 (8–10.8) g/dL. *Msp1* allelic diversity had no noticeable effect on Hb levels (F_6,717_ = 0.87, P = 0.52, Fig. 3a) and there was no difference in Hb levels between single and mixed infections (F_1,720_ = 1.16, P = 0.28, Fig. 3b). In contrast, there was a significant effect of *msp2* allelic diversity (F_2,710_ = 4.84, P = 0.008, Fig. 3c), with patients harboring parasite genotype *msp2*–3D7 in mono-infection showing higher level of Hb than mixed-infected individuals (3D7_FC27) (post-hoc multiple pairwise comparisons: 3D7 *versus* 3D7_FC27, t = 2.7, P = 0.02; 3D7 *versus* FC27, t = 1.7, P = 0.18; FC27 *versus* 3D7_FC27, t = 0.14, P = 0.98). Finally, we found a strong positive relationship between Hb levels and host age (F_1,710_ = 116, P < 0.0001, Fig. 3d), and this effect varied among allelic families (including both mono and mixed infections) of the *msp1* gene (i.e. significant *msp1* by age interaction: F_6,711_ = 2.5, P = 0.02, Fig. 3d). There were no other significant interactions.

## Discussion

Severity and malaria disease outcomes stem from a complex interaction between human host, parasite and environment. Understanding of this interaction is crucial to inform disease case management and treatment decision. Evidence of differences in parasite virulence between different strains has been provided in animal models^8,49^. In this study, the extent to which some specific *P. falciparum* variants are associated with particular malaria symptoms was explored. Merozoite surface antigen variations are among others, more likely to be involved in malaria symptomatology by participating in erythrocyte invasion and immune evasion^49^. Accordingly, we found some significant associations between specific clones of *P. falciparum* and the severity of particular malaria symptoms. In particular, the allelic family *msp1*-MAD20 was associated with highest parasitaemia. In addition, patients harboring *msp2*-FC27 allelic family showed the lowest Hb level in both single and mixed infections. In effect, Soulama *et al*. reported previously that *msp2-*FC27 complexity was associated with severe anaemia during malaria infection^16^. Both high parasitaemia and low Hb level have been known to increase disease severity and for this reason, hyper-parasitaemia and severe anaemia are included among criteria defining severe malaria. This finding would mean that the carriage of *msp1*-MAD20 allelic or *msp2*-FC27 family is associated with increased susceptibility to severe malaria. Several other studies mentioned *msp1*-MAD20 and *msp2*- FC27 allelic families in increasing risk of developing symptomatic malaria or disease severity providing an evidence of association between particular parasite strains and malaria disease outcomes^13,19,50,51^. Body temperature was influenced neither by the *msp1* and *msp2* allelic family variation nor by the multiplicity of distinct parasite strains. In contrast, there was a positive relationship between body temperature and parasite density. This could be explained by cell necrosis due to high level of parasitaemia with elevation of produced cytokines such as tumor necrosis factor (TNF) that mediate malaria fever^52,53^. However, a previous study reported a positive association of co-infections by *msp1*-RO33 + K1 with fever^14^.

Our findings regarding fever and anaemia could be influenced by other pathologies that could cause the same symptoms and this constitutes a limitation of our study. Furthermore, the polymorphic markers (*msp1* and *msp2*) in this study are under immune pressure and this could affect the extent of parasite diversity^50,54^. However, no *msp1* or *msp*2 allele was overrepresented in uncomplicated or severe malaria groups in the univariate analysis. This is probably due to the lack of extremely severe cases such as cerebral malaria in this study, which considered only high parasitaemia and low Hb level as severity criteria.

Except for patients harboring the triple infection K1 + MAD20 + RO33, parasite density decreased with age likely due to the acquisition of natural immunity to malaria in high malaria transmission settings such as Nanoro^55^. Why this relationship was not observed in the case of the triple infection could be explained by the relatively young age of patients belonging to this group compared to other groups (median = 3, min = 1, max = 14 year-old, in K1 + MAD20 + RO33, n = 155; *versus* median = 4, min = 0.4, max = 40 year-old in other groups, n = 570 Fig. 1c). In other words, patients in this group have not yet acquired immunity against circulating parasite haplotypes because they are on average younger and had less chances to encounter the variety of circulating parasites compared to older patients. However, when restricting the statistical analysis to young patients ≤14 year-old for all alleles, we confirmed that overall density decreased with patient age except for the patients harboring the triple infection K1 + MAD20 + RO33 (see Supplementary Information). It is now clear that clinical immunity to malaria is mainly variant-specific^3,56^. It is therefore possible that the presence of multiple and cross-reacting antigens in this triple infection group (K1 + MAD20 + RO33) impaired the immune response in this relatively young age group as previously demonstrated^57^.

Our findings suggest a competition among co-infecting genetically diverse *P. falciparum* within human host leading to attenuation of the growth of parasites belonging to the MAD20 allelic family. As this allelic family seems to be more virulent through its association with hyper-parasitaemia, the high prevalence of mixed-infections could be beneficial in terms of suppressing this virulent strain family. Factors underlying within-host competition are still under assessment but evidence of this phenomenon in human malaria parasites was previously proposed^58^. As age-density relationship was similar in all individual allelic families, underlying a similar relationship with host immune response, we postulate that the observed competition could be mediated by red blood cells resources^59^.

Finally, a large diversity of circulating parasite strains was observed in Nanoro area with frequent occurrence of multiple infections. All the three different allelic families of *msp1* and the two allelic families of *msp2* were represented without significant difference between the two study sites (Nanoro and Nazoanga)^44^. This was likely due to the proximity between the two sites (around 15 km) with possible important population flow from one to the other. Indeed, Nanoro was one of the *RTS,S/AS01* vaccine trial sites and this genetic diversity of circulating parasite clones raises the issue of clone responsiveness i.e. whether different strains observed in our dataset could have responded differently to the vaccine^60^. In bacterial infections such as meningitis, clone differences constituted for a long time one cause of vaccine failure so that parasite clonal diversity must be taken into account in implementing malaria vaccine in order to avoid this phenomenon^61^.

## Conclusion

This study explored the effect of *P. falciparum* genetic diversity on the severity of particular malaria symptoms as measured by parasite density, Hb level and body temperature. While allelic family *msp1* and *msp2* had no significant effect on temperature, we found that parasite density was influenced by the *msp1* allelic family, with highest parasitemia observed for MAD20, followed by RO33 and K1. In addition, clinical isolates containing multiple clones of *P. falciparum* at the *msp1* or the *msp2* genes (i.e. multiple infections) displayed similar parasite density as isolates with monoclonal infections, suggesting the existence of within-host competition. For MAD20, parasite densities significantly decreased in mixed infections with K1, suggesting that in those mixed infections, MAD20 cannot grow to the same levels as it would alone, and hence pointing to the existence of competitive suppression. Finally, there was a significant effect of *msp2* allelic diversity on Hb level with patients harboring parasite genotype *msp2*–3D7 in mono-infection showing higher level of Hb than mixed-infected individuals (3D7_FC27).

## Electronic supplementary material

Supplementary information
