# Supplementary material for: Genetically diverse Plasmodium falciparum infections, within-host competition and symptomatic malaria in humans
Source: Sci Rep. 2019 Jan 15;9:127. doi: 10.1038/s41598-018-36493-y (PMC6333925; doi:10.1038/s41598-018-36493-y)
Supplement: Supplementary file 1 — Supplementary information [file 41598_2018_36493_MOESM1_ESM.pdf]

**Genetically diverse *Plasmodium falciparum* infections, within-host competition and symptomatic malaria in humans.**

Paul SONDO[1*], Karim DERRA[1], Thierry LEFEVRE [1,2], Seydou DIALLO NAKANABO[1], Zekiba TARNAGDA[1], Odile ZAMPA[3], Adama KAZIENGA[1], Innocent VALEA[1,3], Hermann SORGHO[1], Jean-Bosco OUEDRAOGO[1], Tinga Robert GUIGUEMDE[3], and Halidou TINTO[1,3]

1. Institut de Recherche en Sciences de la Santé (IRSS) / Clinical Research Unit of Nanoro (CRUN)

2. MIVEGEC, IRD, CNRS, University of Montpellier, Montpellier, France

3. Centre Muraz of Bobo-Dioulasso, Burkina Faso

*Corresponding author: paulsondo@yahoo.fr

A statistical analysis using a subset consisting of individuals ≤14 year-old (same age range as the K1+MAD20+RO33+ group) on all alleles.

| | Df | Deviance | Resid. Df | Resid. Dev | Pr(>Chi) | |
|---|---|---|---|---|---|---|
| NULL | | | 703 | 908.73 | | |
| Age | 1 | 20.7743 | 702 | 887.95 | 5.167e-06 | *** |
| MSP1 | 6 | 20.5855 | 696 | 867.37 | 0.0021771 | ** |
| MSP2 | 2 | 1.5577 | 694 | 865.81 | 0.4589397 | |
| Age:MSP1 | 6 | 23.1005 | 688 | 842.71 | 0.0007635 | *** |
| Age:MSP2 | 2 | 1.6307 | 686 | 841.08 | 0.4424736 | |
| MSP1:MSP2 | 12 | 19.1153 | 674 | 821.96 | 0.0857834 | . |

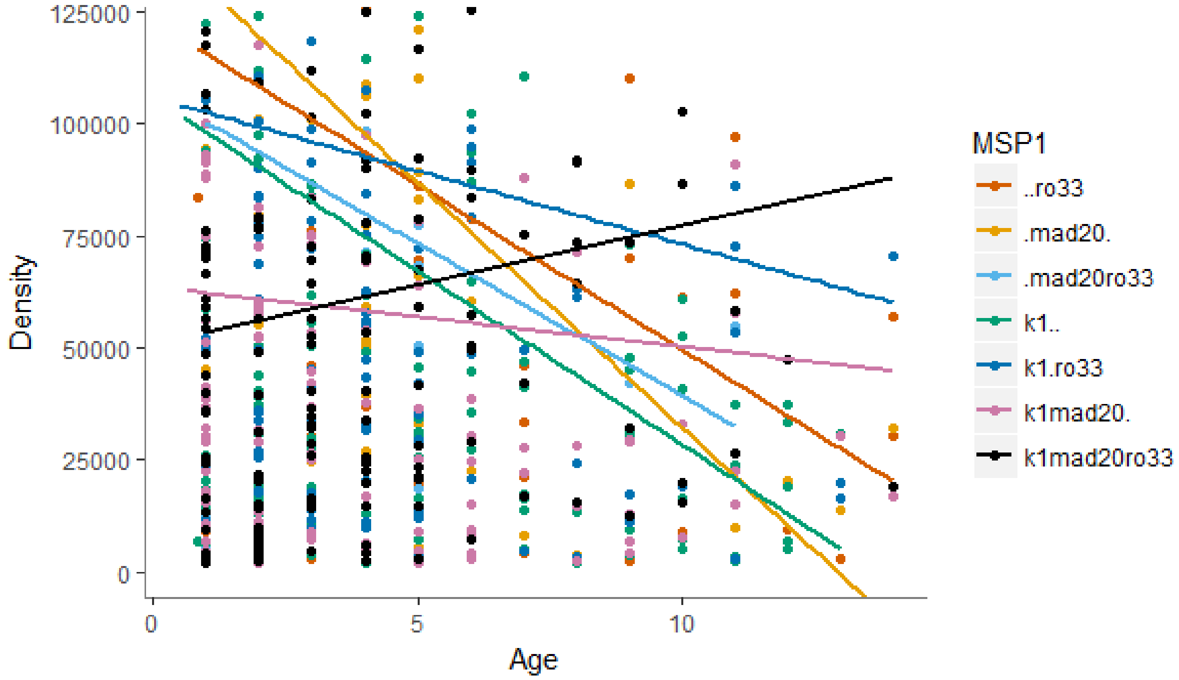


This analysis confirms that: (i) Parasite density was significantly influenced by the *msp1* allelic family with highest parasitaemia observed for MAD20, followed by RO33 and K1; (ii) density decreased with patient age; and (iii) there was a significant age by *msp1* interaction such that parasite density decreased with age except for the patients harboring the triple infection K1+MAD20+RO33.
